# *Escherichia coli* O88 induces intestinal damage and inflammatory response through the oxidative phosphorylation and ribosome pathway in Pekin ducks

**DOI:** 10.3389/fcimb.2022.940847

**Published:** 2022-08-17

**Authors:** Chong Li, Shuzhen Li, Jinmei Liu, Huiyi Cai, Guohua Liu, Xuejuan Deng, Wenhuan Chang

**Affiliations:** ^1^ Key Laboratory for Feed Biotechnology of the Ministry of Agriculture and Rural Affairs, Institute of Feed Research, Chinese Academy of Agriculture Sciences, Beijing, China; ^2^ Precision Livestock and Nutrition Laboratory, Teaching and Research Centre (TERRA), Gembloux Agro-Bio Tech, University of Liège, Gembloux, Belgium; ^3^ Research and Development Department, National Engineering Research Center of Biological Feed, Beijing, China

**Keywords:** Pekin duck, *Escherichia coli*, intestinal barrier, immunity, RNA-seq, oxidative phosphorylation, ribosome

## Abstract

Colibacillosis is one of the major health threats in the poultry industry worldwide. Understanding the pathogenic mechanisms involved in *Escherichia coli*-induced inflammatory response may lead to the development of new therapies to combat the disease. To address this, a total of 96 1-day-old male lean Pekin ducklings were employed and randomly allocated to two treatments, each with six replicates of eight ducks. Ducks in the experiment group (EG) and the control group (CG) were separately orally administered with 0.2 ml of pathogenic *E. coli* O88 (3 × 10^9^ CFU/ml) or equivalent volumes of 0.9% sterile saline solution on day 7, two times with an 8-h interval. Serum and intestinal samples were collected on days 9, 14, and 28. Results showed that ducks challenged with *E. coli* had lower average daily gain and higher feed intake/weight gain during days 9–14 and overall (*P *< 0.05). Histopathological examination showed that *E. coli* decreased the villus height and the ratio of villus height/crypt depth in the jejunum (*P *< 0.05) on days 9 and 14. The intestinal barrier was disrupted, presenting in *E. coli* ducks having higher serum DAO and D-LA on days 9 and 14 (*P *< 0.05) and greater content of serum LPS on day 9 (*P *< 0.05). *Escherichia coli* infection also triggered a systemic inflammatory response including the decrease of the serum IgA, IgM, and jejunal sIgA on day 14 (*P *< 0.05). In addition to these, 1,062 differentially expressed genes were detected in the jejunum tissues of ducks by RNA-seq, consisting of 491 upregulated and 571 downregulated genes. Based on the KEGG database, oxidative phosphorylation and the ribosome pathway were the most enriched. These findings reveal the candidate pathways and genes that may be involved in *E. coli* infection, allow a better understanding of the molecular mechanisms of inflammation progression and may facilitate the genetic improvement of ducks, and provide further insights to tackle the drug sensitivity and animal welfare issues.

## Introduction

*Escherichia coli* (*E. coli*) is a commensal microorganism capable of causing both localized and invasive infections, which is responsible for considerable economic losses in the poultry industry (Kaper et al., 2004). In poultry, pathogenic *E. coli* triggers serious health problems and reduces production, and although it has existed for more than a century, there is still a lack of clarity on its pathogenesis. Ducks are highly susceptible to infections with the bacteria, and the infected ones show progressive debilitation and appear to be thinner, leading to systemic failure and ultimately death (Dziva and Stevens, 2008; Lutful Kabir, 2010; Guabiraba and Schouler, 2015). According to previous reports, the pathogenesis of *E. coli* is complex, but the procedure is similar. The bacteria adhere to and colonize the epithelium of the intestinal tract (in fimbriae/non-fimbrial ways), which induces the disruption of the intestinal epithelial barrier; they also increase gut permeability and enter the bloodstream by crossing the epithelium. Ultimately, *E. coli* secretes various virulence factors, causing inflammatory reactions in various tissues and organs (Peng et al., 2019; Kim et al., 2020; Tan et al., 2020). However, there is little information about the deeper mechanism of *E. coli* infection on intestinal injury and immunity of ducks. In the early stage, our team focused on the negative effect of pathogenic *E. coli* on waterfowl and successfully established an infection model of Pekin ducklings (Liu et al., 2020). In this study, we will further investigate the effects of *E. coli* O88 on duck intestinal health and its possible molecular mechanisms through phenotypic analysis and transcriptomics.

## Materials and methods

The experiment was conducted in Nankou pilot base of the Chinese Academy of Agricultural Sciences (CAAS, Beijing, China). The methods for the animal experiments were set out by the National Institute of Animal Health, and research reporting follows the guidelines of ARRIVE (Kilkenny et al., 2012).

### Animal management

A total of 96 newly hatched, male lean Pekin ducklings were randomly allocated to two treatment groups, each with six replicates of eight ducks. The experiment lasted for 28 days. The ducks were housed in an environmentally controlled facility (fiberglass feeders and plastic net floor), had *ad-libitum* access to feed and water, and exposed to a 16-h light:8-h dark cycle throughout the experiment. Relative humidity was at 60%–70% during days 1–7 and then at 50%–60% for the remainder of the experiment. The ambient temperature was maintained at 33°C ± 2°C during the first week and then gradually decreased to 24°C (1–2°C per day) till the end of the study. The diets were formulated to meet the nutritional requirements of the ducks as determined by the National Research Council (NRC, 1994) and the Nutrient Requirements of Meat-type Duck published by the Ministry of Agriculture of the People’s Republic of China, NY/T 2122-2012 (**Supplementary Table S1**) (National Research Council, 1994; Ministry of Agriculture of the People’s Republic of China, 2004).

### Oral challenge

**Figure 1:**
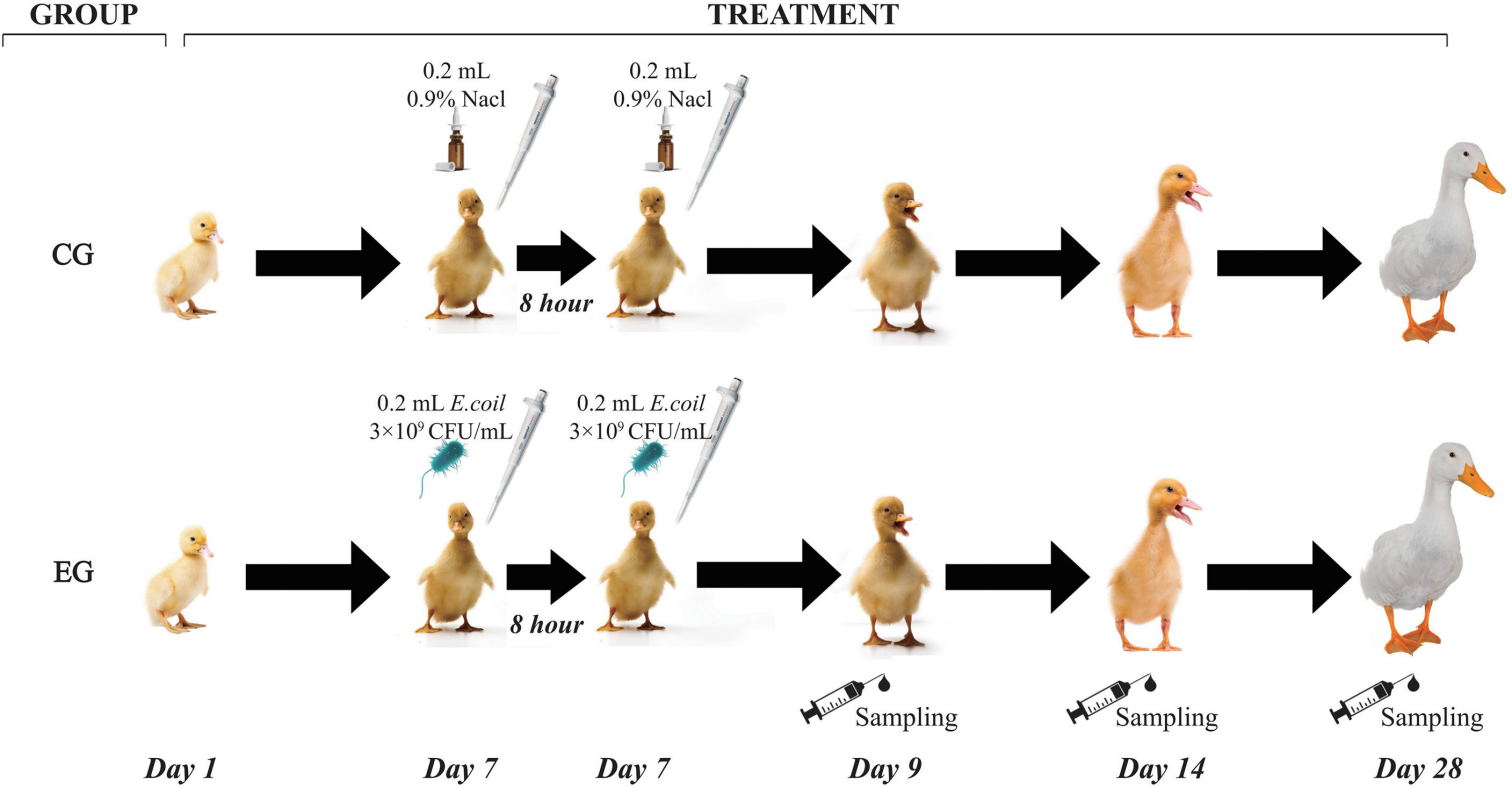
Workflow of the experiment. CG, control group (non-challenged with pathogenic *Escherichia coli* O88); EG, experiment group (challenged with pathogenic *E. coli* O88).

The strain of pathogenic *E. coli* O88 was obtained from China Veterinary Culture Collection Center (CVCC, Beijing, China). The infection model was established based on our previous protocol (Liu et al., 2020). Briefly, the frozen *E. coli* O88 was thawed and cultured in Luria–Bertani (LB) broth to activate three times (37°C, 12 h). *Escherichia coli* O88 was resuspended in a sterilized 0.9% saline solution and counted by plate cultivation. On day 7, all ducks of the experiment group (EG) were orally administered with 0.2 ml of *E. coli* O88 (3 × 10^9^ CFU/ml) twice, 8 h apart. The control group (CG) was orally administered with equivalent volumes of 0.9% sterile saline solution, and the workflow is shown in **Figure 1**.

### Sampling

On days 9, 14, and 28 following 12 h of fasting, all ducks were weighed, and feed intake was measured on a per cage basis. Average daily feed intake (ADFI), average daily gain (ADG), and the feed intake/weight gain ratio (F/G ratio) were calculated, and mortality was recorded as the percentage of the ducks culled and found dead. One duck close to the average body weight (BW) from each replicate was selected. Blood samples were taken (2.5 ml) from the wing vein using an anticoagulant-free vacuum test tube (5 ml) and immediately placed on ice. Serum was harvested after centrifuging at 3,000×*g* for 10 min and stored at −20°C until analyzed. The ducks were euthanized by electric stunning and immediately manually slaughtered. After opening longitudinally and flushing the residual digesta with ice-cold phosphate-buffered saline (PBS), the middle portion of the jejunal tissue (1.5 cm) was collected, extracted, and fixed in 10% neutral formalin to analyze intestinal mucosal morphology (Watkins et al., 2004). The jejunal mucosa was directly scraped using a sterile glass microscope slide at 4°C for the analysis of secretory immunoglobulin A (sIgA) levels. The jejunal tissue and mucosa were snap-frozen in liquid nitrogen and transferred to a −80°C freezer until analyzed.

### Intestinal morphology

Referring to the method of Lin et al. (2017), the intestinal tissues were fixed in formalin, embedded in paraffin, deparaffinized, dehydrated, and stained. Observing the morphological structure of the jejunum by a microscope (ML51 MSho, Guangzhou, China) and analyzing by a digital imaging system (Msho), villus height and crypt depth were measured in at least 10 well-oriented villi and then the villus height/crypt depth ratio (V/C ratio) was calculated.

### Serum indices

The serum diamine oxidase (DAO, Kit No. A088-1-1), D-lactic acid (D-LA, Kit No. H263-1-2), and endotoxin lipopolysaccharide (LPS, Kit No. E039-1-1) were measured using commercial kits (Nanjing Jiancheng Bioengineering Institute, Nanjing, China). Immunoglobulin A (IgA, Kit No. E027-1-1), immunoglobulin G (IgG, Kit No. E026-1-1), and immunoglobulin M (IgM, Kit No. E025-1-1) were analyzed by immunoturbidimetry with commercial kits (Nanjing Jiancheng Bioengineering Institute, Nanjing, China).

### The content of sIgA in intestinal mucosa

The sIgA content of the jejunal mucosa was determined by referring to the method of Alhotan et al. (2021). Jejunal mucosa samples were homogenized, diluted with frozen PBS, and then centrifuged at 4,450×*g* for 15 min in a refrigerated centrifuge to collect the supernatants. The levels of total protein (Tp, Kit No. MBS165636) and sIgA (Kit No. MBS737239) were analyzed using commercial ELISA kits (MyBioSource Inc., San Diego, USA).

### RNA-seq analysis of the jejunum

Total RNA was extracted from jejunum tissues using TRIzol kits (Invitrogen, Carlsbad, CA, USA) and qualitatively and quantitatively analyzed using Agilent 2100 (Agilent Technologies, Inc., Palo Alto, CA, USA). RNA-seq libraries were generated using NEBNext Ultra RNA Library Prep Kit for Illumina (NEB, Ipswich, MA, USA). Libraries were sequenced on a HiSeq2500 platform (Illumina Inc., San Diego, CA, USA) to generate paired-end 100-bp raw reads.

The adaptor sequences and low-quality sequence reads were removed from the data sets using the FASTX-Toolkit tool. The clean reads were then mapped to the reference genome sequence. Gene expression levels were quantified as fragments per kilobase of transcript per million (FPKM) mapped from different jejunum samples (Florea et al., 2013). Differential expression analysis of the CG and EG was performed using the DESeq2 R package (1.6.3) (Love et al., 2014). Genes with the parameter of *P*-value <0.1 and log_2_ (fold-change) ≤0.5 or log_2_ (fold-change) >0.5 as determined by DESeq2 were considered differentially expressed. Differentially expressed genes (DEGs) were submitted for annotation and enrichment analyses using the Kyoto Encyclopedia of Genes and Genomes (KEGG, http://www.kegg.jp/eg/pathway.html) using the clusterProfiler R package (3.10.1).

### RT-qPCR

The six DEGs, namely, ribosomal protein S28 (*RPS28*, Accession: XM_027471935.2), mitochondrial ribosomal protein L23 (*MRPL23*, Accession: XM_027458708.2), ribosomal protein L34 (*RPL34*, Accession: XM_027456389.2), ATP synthase membrane subunit g (*ATP5MG*, Accession: XM_027444122.2), cytochrome c oxidase subunit 7B mitochondrial (*COX7B*, Accession: XM_027465058.2), and ATP synthase membrane subunit F (*ATP5MF*, Accession: XM_027468823.2), were randomly selected, and RT-qPCR was used to confirm the accuracy and reliability of the RNA-Seq results. The total RNA from the jejunum tissues was isolated using TRIzol reagent (TIANGEN, Beijing, China) and reversely transcribed into complementary DNA (cDNA) pursuant to the manufacturer’s protocol. The concentration of total RNA was determined from OD 260/280 with a spectrophotometer (Ultrospec 2100 pro, GE Healthcare, Piscataway, USA), and purity was measured by agarose gel electrophoresis. Then, 500 ng of total RNA was reverse-transcribed into cDNA using the PrimeScript of Fast Quant RT Kit (with gDNase) (TIANGEN, Beijing, China) according to the manufacturer’s protocol. qPCR was conducted using the Biosystems Bio-Rad Real-Time PCR system (Bio-Rad, Carlsbad, CA, USA) with the Brilliant SYBR Green qPCR Master Mix (Stratagene, La Jolla, CA, USA). The primers used are listed in **Table 1**. *β-Actin* was used to normalize the expression of the targeted genes. The mRNA level of the relative gene was calculated using the 2^−ΔΔCt^ method (Livak and Schmittgen, 2001). All the samples were analyzed in triplicate using the geometric mean of internal references.

**Table 1:** Primers used for the RT-qPCR in this study.

Genes	Accession number	Primer sequence (5′–3′) Sense/antisense forward primer	Production length (bp)
*RPS28*	XM_027471935.2	Forward: CTCCATCATCCGCAACGTGA	107
		Reverse: AGTCTTCAGCACCGGCTCAG	
*MRPL23*	XM_027458708.2	Forward: GCTCCCAGTACAGCAGAATGA	122
		Reverse: GTGGTTCCTCTTGTTGTTCGC	
*RPL34*	XM_027456389.2	Forward: AGGGAAGGCACCAAAGTCAG	169
		Reverse: AGCTCGCTTGATCCTGTCG	
*ATP5MG*	XM_027444122.2	Forward: AGATCACCGTCAAGGAAGCAC	143
		Reverse: CAGCAAACAGCGTAACTAAGG	
*COX7B*	XM_027465058.2	Forward: CATGAGCCAAACTTCCATGACA	95
		Reverse: GCCTGCGTGAACACATAACC	
*ATP5MF*	XM_027468823.2	Forward: AGAGACACACCCAAGATGGC	242
		Reverse: TACCTCTCATACCCTCTGCG	
*β-actin*	EF667345.1	Forward: GCTATGTCGCCCTGGATTT	160
		Reverse: GGATGCCACAGGACTCCATAC	

### Statistical analysis

The data were analyzed by a one-factor ANOVA procedure of the SPSS 19.0 software package for Windows (SPSS Inc., Chicago, IL, USA). Significant differences between treatment means were separated using Duncan’s multiple range test. Differences were considered significant at *P <*0.05. The graphs were designed using GraphPad Prism 9 Project (GraphPad Software Inc., San Diego, CA, USA) and Origin 8.5 (OriginLab, Berkeley, CA, USA).

## Results

### Growth performance

The growth performance of the ducks is shown in **Table 2**. *Escherichia coli* infection significantly decreased the BW of the ducks on days 14 and 28 (*P *< 0.05) and the ADG during days 9–14 and overall. In addition, the challenge increased the F/G ratio during days 9–14 and overall (*P *< 0.05). However, there was no significant difference in ADFI. Furthermore, the *E. coli* challenge also resulted in serious mortality, while in the CG, it was relatively low during the experiment (*P *< 0.05).

**Table 2:** Effect of the *Escherichia coli* challenge on the growth performance of ducks (*n* = 6).

Parameter	Days	Treatment		SEM	*P*-value
		CG	EG		
BW, g	9	301.7	290.1	4.256	0.259
	14	600.5	557.5	10.441	0.031
	28	1,690.1	1,558.1	3.091	0.024
ADG, g/day/duck	1–9	27.55	27.12	0.506	0.693
	9–14	59.76	52.75	1.723	0.034
	14–28	77.50	72.38	1.487	0.084
	1–28	58.56	53.84	1.107	0.024
ADFI, g/day/duck	1–9	35.49	36.34	0.586	0.493
	9–14	77.19	74.44	1.776	0.466
	14–28	134.67	137.68	2.506	0.574
	1–28	90.15	92.20	1.430	0.500
F/G ratio	1–9	1.29	1.34	0.017	0.137
	9–14	1.30	1.41	0.033	0.047
	14–28	1.74	1.91	0.045	0.051
	1–28	1.54	1.72	0.043	0.026
Mortality, %	28	4.17	16.67	2.590	0.007

BW, body weight; ADG, average daily gain; ADFI, average daily feed intake; F/G ratio, feed intake/weight gain; CG, control group (non-challenged with pathogenic E. coli O88); EG, experiment group (challenged with pathogenic E. coli O88).

### Intestinal morphology

**Figure 2:**
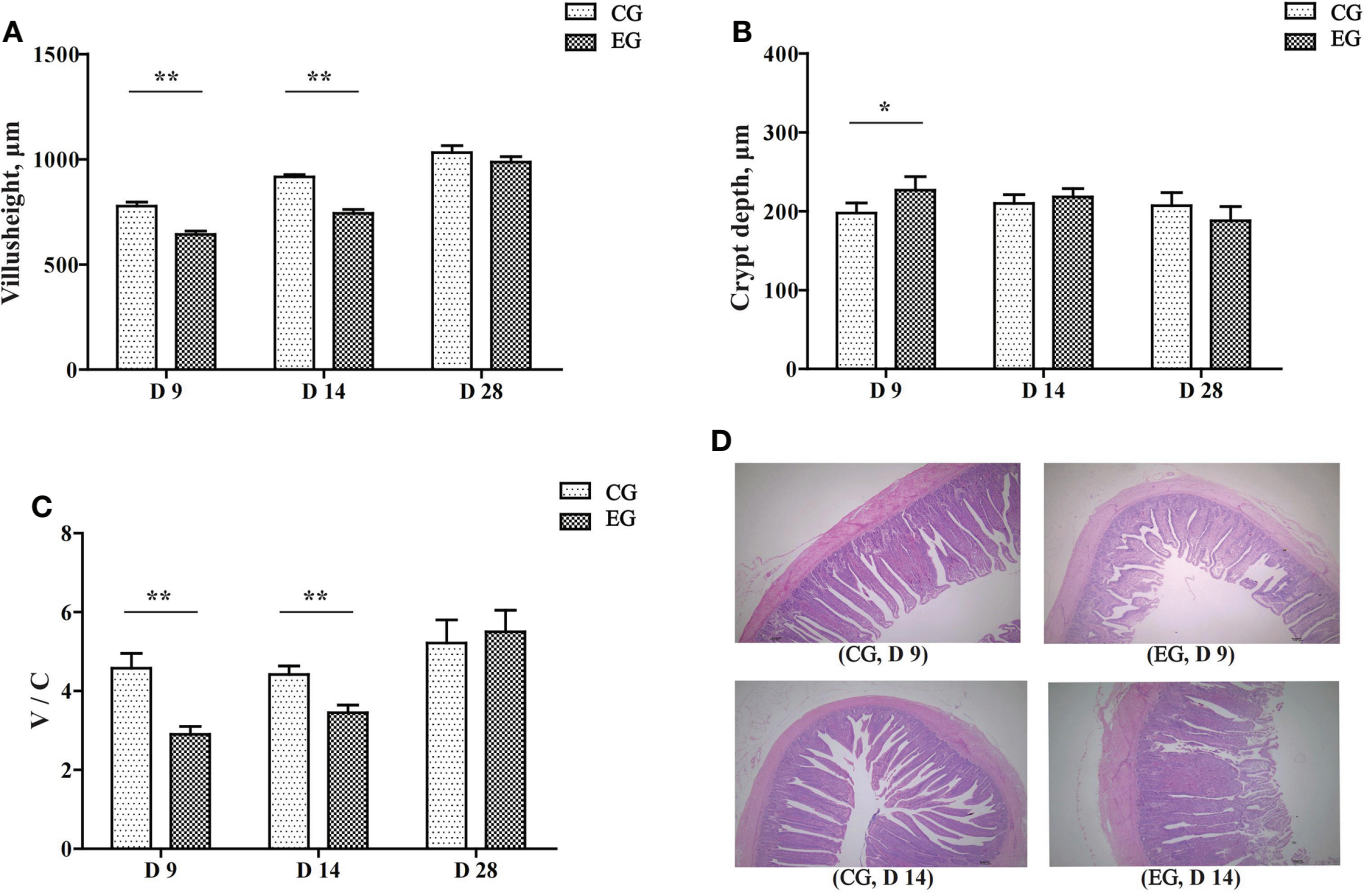
Effect of the *Escherichia coli* challenge on the jejunum histomorphology of the ducks. CG, control group (non-challenged with pathogenic (*E*) *coli* O88); EG, experiment group (challenged with pathogenic (*E*) *coli* O88). **(A)** Villus height in the jejunum of ducks; **(B)** crypt depth in the jejunum of ducks; **(C)** the ratio of villus height/crypt depth in the jejunum of ducks; **(D)** histological damage in the jejunum of ducks on days 9 and 14 (normal, villi shortened, damaged, and eroded). Data are indicated as means ± SEM (*n* = 6), * 0.01 < *P* < 0.05; **P < 0.01.

**Figure 2** represents the intestinal morphological analysis of the jejunum. On days 9 and 14, the villus height of the EG ducks was significantly decreased (*P* < 0.01, **Figure 2A**). The crypt depth of the EG ducks was significantly increased on day 9 (*P* < 0.05, **Figure 2B**). The jejunal V/C ratio was significantly decreased in the EG ducks on days 9 and 14 (*P* < 0.01, **Figure 2C**). **Figure 2D** displays the morphology changes of the jejunum on days 9 and 14. The jejunal villi of the CG ducks had finger-like protrusions and were well extended, arranged neatly, clear, and full, whereas the jejunal villi of the EG ducks were disorganized and interrupted. The intestinal mucosa appeared atrophic and necrotic, and the integrity of the chorionic epithelium was seriously disrupted.

### Intestinal permeability parameters

As presented in **Table 3**, the *E. coli* challenge significantly increased the concentration of serum DAO on days 9, 14, and 28 (*P *< 0.05), and the concentration of D-LA also increased significantly on days 9 and 14 in the EG ducks (*P *< 0.05). The serum LPS level in the *E. coli* challenge ducks was increased on day 9 significantly (*P *< 0.05), but there was no significant difference on days 14 and 28 between the two groups.

**Table 3:** Effect of the *Escherichia coli* challenge on the serum DAO, D-LA, and LPS content of ducks (*n* = 6).

Parameter	Days	Treatment		SEM	*P-*value
		CG	EG		
DAO (U/L)	9	1.35	1.58	0.059	0.039
	14	1.63	1.88	0.053	0.008
	28	1.33	1.58	0.314	0.008
D-LA (μmol/L)	9	4.25	5.42	0.261	0.016
	14	2.94	4.54	0.372	0.023
	28	3.86	3.92	0.229	0.889
LPS (EU/mL)	9	0.56	0.63	0.013	0.003
	14	0.58	0.62	0.009	0.073
	28	0.66	0.68	0.012	0.463

DAO, diamine oxidase; D-LA, D-lactic acid; LPS, lipopolysaccharide; CG, control group (non-challenged with pathogenic E. coli O88); EG, experiment group (challenged with pathogenic E. coli O88).

### Immune parameters in *the* serum and jejunal mucosa

**Figure 3:**
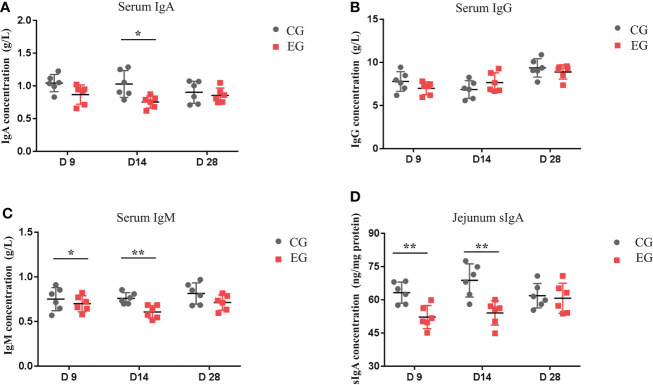
Effect of the *Escherichia coli* challenge on immune factors in the serum and intestine of the ducks. CG, control group (non-challenged with pathogenic (*E*) *coli* O88); EG, experiment group (challenged with pathogenic (*E*) *coli* O88). **(A)** Serum IgA, immunoglobulin A; **(B)** serum IgG, immunoglobulin G; **(C)** serum IgM, immunoglobulin M; **(D)** jejunum sIgA, secretory immunoglobulin **(A)** Data are indicated as means ± SEM (*n* = 6), * 0.01 < *P* < 0.05; **P < 0.01.

**Figure 3** represents the serum and jejunal mucosa immune parameters of the ducks, and there was a similar variation tendency of the serum IgA and IgM levels. The level of serum IgA was lower in the EG compared to the CG on day 14 (*P* < 0.05), and the level of serum IgM in the EG was significantly lower on days 9 and 14 (*P* < 0.05 and *P* < 0.01). Furthermore, the EG showed a lower sIgA level in the jejunal mucosa than the CG on days 9 and 14 (*P* < 0.01). *Escherichia coli* infection did not affect the serum IgG level.

### Sequencing the *transcriptome and aligning and mapping reads to the g*enome

To define the mechanism of the *E. coli* challenge, 12 sequencing libraries, namely, the CG (CG-1, CG-2, CG-3, CG-4, CG-5, CG-6) and the EG (EG-1, EG-2, EG-3, EG-4, EG-5, EG-6), were constructed. After quality control of the sequencing data, 20,587,116 to 36,968,656 clean reads were obtained to establish 12 RNA-seq libraries. The clean data of each sample reached 6.14 GB, the GC content ranged from 50.45% to 52.09%, and the percentage of the Q30 base was above 94.44%. Sequence alignment of each sample was conducted against the designated reference genome, with efficiency from 65.01% to 73.58%, and the statistics of the sequencing data are shown in **Table 4**.

**Table 4:** Characteristics of the reads from the jejunum libraries of 12 ducks.

Samples ID	Clean reads	Clean bases	GC content%	≥Q30%	Mapped reads	Uniq mapped reads
CG-1	27,151,835	8,110,973,874	51.75	94.85	37,671,999 (69.37%)	36,816,201 (67.80%)
CG-2	27,592,676	8,252,157,462	51.24	95.05	35,874,665 (65.01%)	34,886,867 (63.22%)
CG-3	36,968,656	11,052,522,666	52.09	95.15	50,496,317 (68.30%)	49,161,348 (66.49%)
CG-4	31,021,027	9,275,561,318	51.60	95.22	44,667,025 (71.99%)	43,515,483 (70.14%)
CG-5	25,794,423	7,711,780,046	52.02	95.04	35,154,583 (68.14%)	34,369,477 (66.62%)
CG-6	34,562,790	10,321,923,262	52.07	94.74	47,991,258 (69.43%)	46,658,989 (67.50%)
EG-1	20,587,116	6,140,986,574	50.45	95.07	30,216,876 (73.39%)	29,592,927 (71.87%)
EG-2	26,608,953	7,945,804,612	51.41	95.34	37,457,259 (70.38%)	36,610,632 (68.79%)
EG-3	30,147,965	9,017,205,702	51.88	94.67	43,579,994 (72.28%)	42,455,696 (70.41%)
EG-4	31,570,825	9,433,393,492	51.96	94.50	43,391,416 (68.72%)	42,298,779 (66.99%)
EG-5	29,190,041	8,715,512,362	51.83	94.60	42,953,175 (73.58%)	41,773,866 (71.55%)
EG-6	30,843,988	9,213,607,330	51.72	94.44	43,547,846 (70.59%)	42,561,526 (68.99%)

Clean reads, counts of clean PE reads; Clean bases, total base number of clean data; GC content, percentage of G,C in clean data; ≥Q30%, percentage of bases with Q-score no less than Q30; Mapped reads, counts of mapped reads and their proportion in clean data; Uniq mapped reads, counts of reads mapped to a unique position on the reference genome and their proportion in clean data; CG, control group (non-challenged with pathogenic E. coli O88); EG, experiment group (challenged with pathogenic E. coli O88).

### DEG analysis

**Figure 4:**
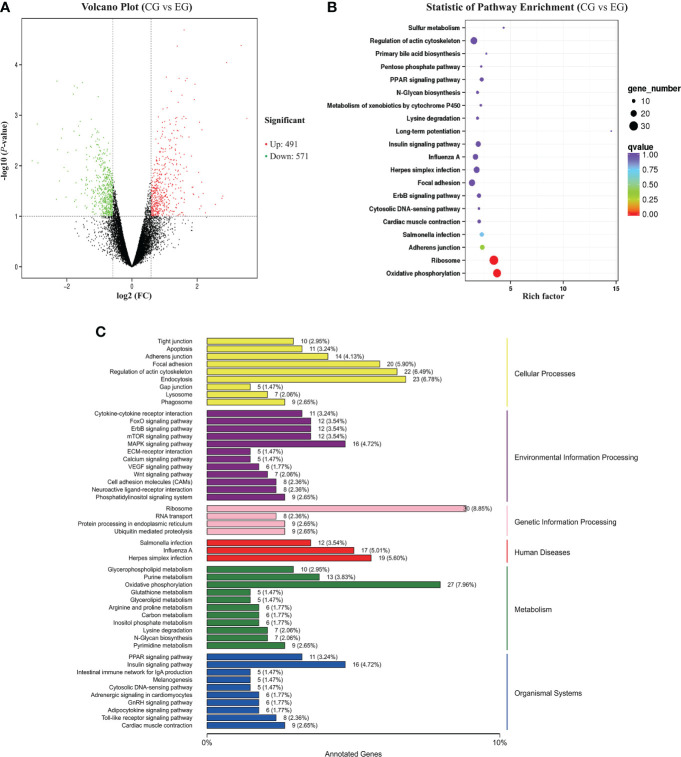
RNA-seq analysis of the jejunum tissue of the ducks (CG vs. EG). CG, control group (non-challenged with pathogenic (*E*) *coli* O88); EG, experiment group (challenged with pathogenic (*E*) *coli* O88). **(A)** Volcano plot of differentially expressed genes. The *x*-axis shows the log2 (FC) of DEGs between the two groups, and the *y*-axis indicates the negative logarithm of the *P*-value; each point in the volcanic map represents a gene; red represents increased expression, while green represents decreased expression. **(B)** Dot plot of the KEGG pathway enrichment analysis of differentially expressed genes. **(C)** KEGG classification of differentially expressed genes. The vertical axis is the name of the KEGG pathway. The horizontal axis is the number of genes and the proportion of genes from the total annotated genes.

The DEGs between the CG and EG were screened according to a fold change of ≥2 and a false discovery rate (FDR) of <0.05. FPKM was applied to determine the expression level of a gene. Volcano plots of transcriptome sequencing data were used to visualize the distribution of DEGs between the two groups. Between the CG and EG, 1,062 genes were differentially expressed, consisting of 491 upregulated and 571 downregulated genes (**Figure 4A**).

### Functional analysis of differentially expressed genes

To better understand the regulatory network of the jejunum damage caused by *E. coli*, we performed a KEGG pathway enrichment analysis for the 1,931 DEGs detected, and a scatter plot of the KEGG data was created by selecting the top 20 enriched pathways (**Figure 4B**). Among the pathways, marked oxidative phosphorylation (ko00190) and ribosome (ko03010) were the most significant predictors (*P* < 0.01). The DEGs and the variation trend of the top 5 pathways are listed in **Table 5**. All DEGs involved in oxidative phosphorylation (27 DEGs, 7.96%) and ribosome (30 DEGs, 8.85%) pathway were downregulated. The KEGG annotations of DEGs were classified according to the type of cellular processes, including environmental information processing, genetic information processing, human disease, metabolism, and organismal system pathways (**Figure 4C**). The expression values were measured in FPKM, and the key DEGs with known functions contained in these pathways are listed in **Supplementary Figure S1** and **Supplementary Figure S2**, respectively. Compared with the CG, the *E. coli* challenge decreased the expression of oxidative phosphorylation metabolism, and these include, but are not limited to, NADH-ubiquinone oxidoreductase core subunit, mitochondrial cytochrome c oxidase subunit, and ATP synthase membrane subunit. In addition, regarding the signaling pathways of the ribosome, the expression of genes associated with mitochondrial ribosome proteins, ribosome biogenesis, mitochondrial translation factors, etc. was also downregulated. These findings indicated that *E. coli* destroys the jejunum tissue primarily by interfering with energy metabolism and ribosome biogenesis.

**Table 5:** The top 5 KEGG pathway enrichment analysis of DEGs.

KEGG ID	Description	Gene ratio (%)	Enrich factor	*P*-value	*Q*-value	Gene number	Trend
ko00190	Oxidative phosphorylation	7.96	3.74	<0.001	<0.001	27	Down
ko03010	Ribosome	8.85	3.44	<0.001	<0.001	30	Down
ko04520	Adherens junction	4.13	2.34	0.002	0.322	14	Up and down
ko05132	Salmonella infection	3.54	2.30	0.005	0.767	12	Up and down
ko04720	Long-term potentiation	0.29	14.54	0.069	1.000	1	Down

KEGG ID, ID in Kyoto Encyclopedia of Genes and Genomes identity; Gene ratio, the proportion of DEGs associated with the pathway; Enrich factor, ratio of gene numbers to all gene numbers annotated in the KEGG pathway; Q-value, P-value adjusted by the method of Benjamini and Hochberg; Gene number, the number of DEGs in each KEGG pathway; Trend, expression alterations of genes associated with various pathways.

### Validation of gene expression by using RT-qPCR

**Figure 5:**
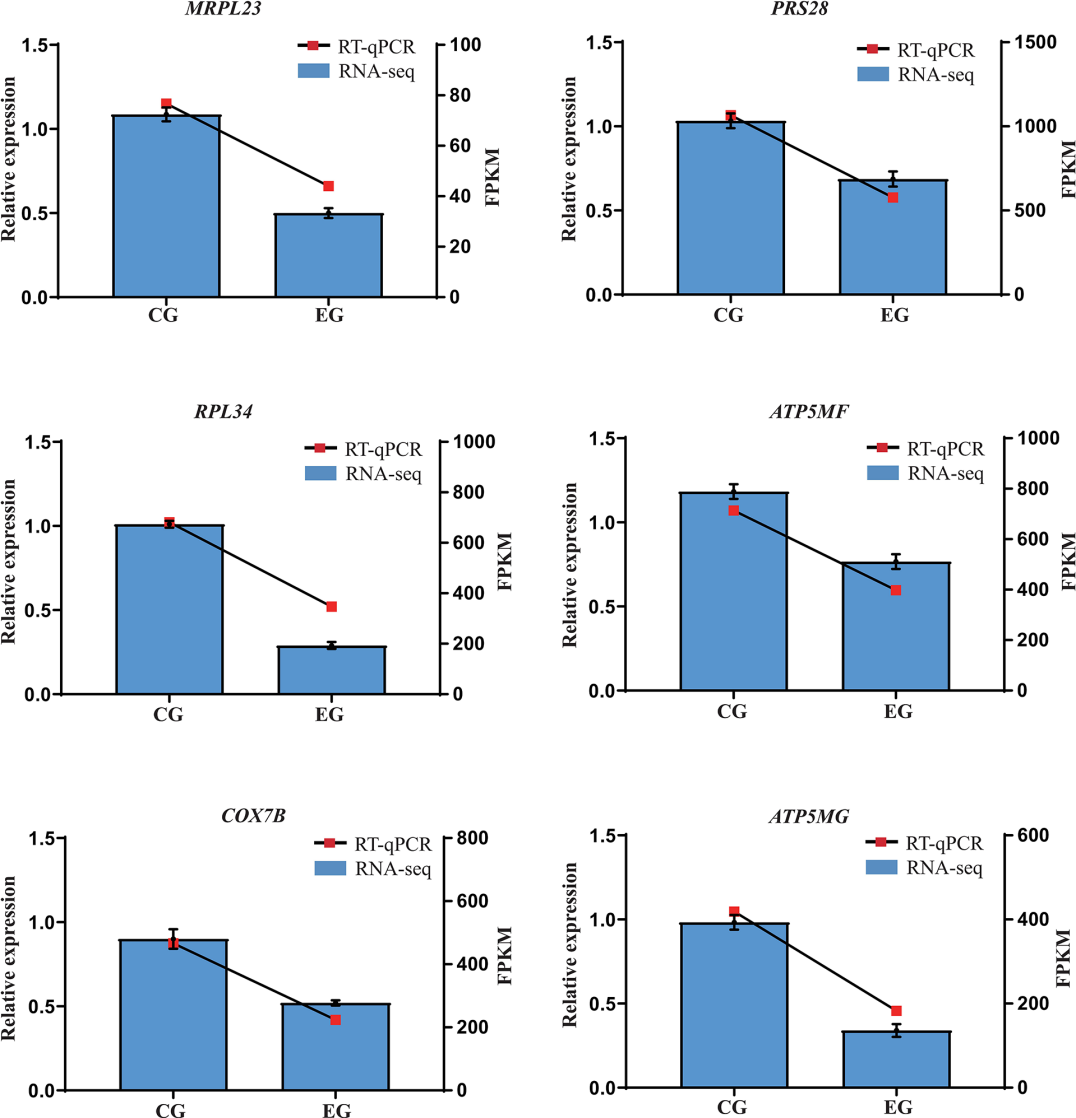
Relative expression levels from RT-qPCR. CG, control group (non-challenged with pathogenic *E. coli* O88); EG, experiment group (challenged with pathogenic *E. coli* O88). Data are indicated as means ± SEM (*n* = 6). The relative expression values were normalized to the *β-actin* gene. *RPS28*, ribosomal protein S28; *MRPL23*, mitochondrial ribosomal protein L23; *RPL34*, ribosomal protein L34; *ATP5MG*, ATP synthase membrane subunit g; *COX7B*, cytochrome c oxidase subunit 7B mitochondrial; *ATP5MF*, ATP synthase membrane subunit F.

Six genes involved in pathways of oxidative phosphorylation (*ATP5MF*, *COX7B*, *ATP5MG*) and ribosome (*MRPL23*, *PRS28*, *RPL34*) were selected for validation by RT-qPCR. Their results show that, compared with the CG, *E. coli* decreased the expression of these selected genes to varying degrees. The changes in the relative expression levels of the RT-qPCR showed similar trends with the transcriptome sequencing analyses (**Figure 5**), suggesting the reproducibility and reliability of previous RNA-seq data.

## Discussion

Colibacillosis is the most common infectious bacterial disease of poultry, which causes serious damage to host health and production loss. Our results showed that *E. coli* O88 significantly decreased the body weight gain and feed conversion rate of ducks. This is consistent with previous studies that showed that *E. coli* infection reduced productivity by threatening host health (Liu et al., 2020; Wu et al., 2021).

The absorption site of nutrients is mainly in the small intestine, especially the villus. The villus height, crypt depth, and the V/C ratio are the gold standards for assessing the health status of the intestinal barrier in poultry (Ducatelle et al., 2018). The height of the villus indicates the area of intestinal absorption (Daneshmand et al., 2019). The depth of the crypt shows the production speed and maturation rate of intestinal epithelial cells (Pédron et al., 2012). The V/C represents the functional state of the intestine (Inatomi and Otomaru, 2018). Our results showed that *E. coli* infection severely disrupted the morphological structure of the jejunum in the early stage and affected nutrient absorption, thereby resulting in lower weight gain and feed conversion efficiency. Intestinal epithelial cells are the target of pathogenic *E. coli*. The endotoxin secreted by the pathogenic bacterium could trigger intestinal inflammation, resulting in massive cell detachment and loss of tight connections leading to a reduction in the intestinal villi height and an increase in crypt depth, thus damaging the intestinal morphological structure (Guabiraba and Schouler, 2015; Berger et al., 2017; Ekim et al., 2020).

Intestinal permeability is a key factor in judging the integrity of the intestinal barrier, and its main biomarkers are DAO, D-LA, and LPS (Ducatelle et al., 2018). DAO is an enzyme that catalyzes the oxidation of diamines, which exists only in the intestinal mucosa and ciliated cells, and its activity reflects the integrity and maturity of the intestinal mucosa (Luk et al., 1980). It has previously been shown that the level of DAO in serum activity is negatively correlated with intestinal permeability. Under normal conditions, it maintains a lower level of concentration. When the intestinal mucosal barrier function is destroyed, the damaged intestinal mucosal epithelial cells release DAO, which elevated the levels of this marker in blood plasma (Honzawa et al., 2011). D-LA is produced by the fermentation and metabolism of intestinal bacteria. It is rarely absorbed under normal physiological conditions; however, when the intestinal mucosal barrier is impaired, inflammation is induced, the permeability of the intestinal mucosa increases, and a large amount of D-LA will enter the blood circulation through the damaged intestinal mucosa (Sun et al., 2001). LPS is the gram-negative bacterial cell wall component (Tomlinson and Blikslager, 2004); it would attack the host innate immune system once absorbed into the intestinal epithelium and seriously damage the immune system of the host (Smith et al., 2010; Stephens and von der Weid, 2020). In this study, we found that pathogenic *E. coli* increased the levels of DAO, D-LA, and LPS in the serum, suggesting a breakdown of the intestinal barrier. In addition, D-LA and LPS components would subsequently leak into the bloodstream through the damaged intestinal mucosa (Qu et al., 2020), providing opportunities for the invasion of harmful metabolites and pathogens, which trigger a robust systemic inflammatory response (Leng et al., 2016; Letsinger et al., 2020; Franciotti et al., 2021).

The sIgA secreted by epithelial immune cells has an immunoprotective function and is considered the first line of defense in mucosal immunity (Li et al., 2020). Our study showed that *E. coli* attacked the immune system by decreasing serum immunoglobulins and interfering with the secretion of jejunum sIgA, which is consistent with another *E. coli* infection study (Alber et al., 2021).

In recent years, the central role of cellular energy metabolism in regulating intestinal inflammatory diseases has received increasing attention (Buck et al., 2016; O’Neill and Pearce, 2016). It has been demonstrated that *E. coli* infection on ducks causes damage to the intestinal barrier and subsequently triggers mucosal inflammatory responses, which is an energy-consuming process (Pi et al., 2014). The mitochondrion plays a central role in energy metabolism homeostasis through the respiratory chain, and ATP synthesis takes place in the mitochondria by oxidative phosphorylation (Ashton et al., 2018; Fujiwara et al., 2021). Our results showed that oxidative phosphorylation complexes downregulated the genes and reduced the mitochondrial oxidative phosphorylation activity in jejunum tissue, resulting in impaired mitochondrial function by *E. coli* infection. This was similar to the report that showed that the activities of jejunal mitochondrial respiratory complexes and the level of ATP were reduced after intestinal damage (Zhang et al., 2021). The mechanism was also demonstrated at the cellular level: LPS-activated macrophages are characterized by high glycolysis and low oxidative phosphorylation (Vats et al., 2006; Rodríguez-Prados et al., 2010), and as a key component of macrophage polarization, these changes contribute to a kind of tissue self-repair in inflammation (Ip et al., 2017; Carrasco-Pozo et al., 2020). Inflammation and metabolic diseases are associated with mitochondrial dysfunction. Mitochondrial oxidative phosphorylation contributes to the production of enough ATP for the body to use (Xiong et al., 2021), and once this process is disrupted, ATP synthesis is also affected (Czarny et al., 2018).

The results of the RT-qPCR verified that *E. coli* infection downregulated the expression of the ATP synthase subunit and cytochrome c oxidase subunit genes, which indicated that the infection interfered with the function of the respiratory chain complex subunits, affecting the assembly of the respiratory chain complex and ultimately leading to mitochondrial oxidative phosphorylation dysfunction. Similar findings have been reported that LPS downregulated the expression of NADH dehydrogenase subunit 1 (ND1), ATP synthase F0 subunit 6 (ATP6), and cytochrome c oxidase subunit 1 (COX1) and induced jejunum mitochondrial damage (Sun et al., 2020). Beyond this, this is consistent with previous reports showing that pathogenic factors induced intestinal mitochondrial dysfunction and damaged the oxidative phosphorylation activity of intestinal epithelial cells; the mechanisms all focused on the downregulation of gene expression of subunits associated with ATP and NADH, ultimately leading to impaired intestinal barrier (Monternier et al., 2015; Sun et al., 2020).

All components of the oxidative phosphorylation-related enzymes are synthesized in mitochondrial ribosomes, which are essential in the regulation of cellular metabolism (Kim et al., 2017). In this study, we also found that *E. coli* infection downregulated the expression of genes encoding for mitochondrial ribosome proteins, ribosome biogenesis, and mitochondrial translation factors, indicating that *E. coli* could destroy the jejunum tissue through intervention with the ribosome and energy metabolism.

## Conclusion

In conclusion, pathogenic *E. coli* O88 infection damaged the integrity of the intestinal epithelium, triggered inflammatory responses, and impaired the growth performance of the ducks by interfering with the energy metabolism pathway and downregulating the gene expression related to oxidative phosphorylation and ribosome. Our findings will help us to further explore the targets for blocking or treating *E. coli* infections and perhaps facilitate the development of vaccines or therapeutic interventions.

## Data availability statement

The datasets presented in this study can be found in online repositories. The names of the repository/repositories and accession number(s) can be found in the article/**Supplementary Material**.

## Ethics statement

The animal study was reviewed and approved by Animal Ethics Committee of the Chinese Academy of Agricultural Sciences.

## Author contributions

CL, WC, and SL: conceptualization. CL, WC, and GL: methodology. CL and HC: investigation, software, data curation, and writing—original draft preparation. JL and XD: validation and resources. CL and SL: formal analysis. CL, SL, and JL: writing—review and editing and supervision. WC: project administration and funding acquisition. The final manuscript has been read and approved by all authors.

## Funding

This research was funded by the Agricultural Science and Technology Innovation Program (ASTIP) and the Collaborative Innovation Task in Agricultural Science and Technology Innovation Program (CAAS-XTCX).

## Conflict of interest

The authors declare that the research was conducted in the absence of any commercial or financial relationships that could be construed as a potential conflict of interest.

## Publisher’s note

All claims expressed in this article are solely those of the authors and do not necessarily represent those of their affiliated organizations, or those of the publisher, the editors and the reviewers. Any product that may be evaluated in this article, or claim that may be made by its manufacturer, is not guaranteed or endorsed by the publisher.

## Supplementary material

Supplementary Figure 1 A KEGG map of the oxidative phosphorylation pathway of DEGs in CG vs EG. CG=control group, non-challenged with pathogenic *E. coli* O88; EG=experiment group, challenged with pathogenic *E. coli* O88. The boxes with green frames correspond to the down-regulation of DEGs in the EG ducks.

Supplementary Figure 2 A KEGG map of the ribosomal pathway of DEGs in CG vs EG. CG=control group, non-challenged with pathogenic *E. coli* O88; EG=experiment group, challenged with pathogenic *E. coli* O88. The boxes with green frames correspond to the down-regulation of DEGs in the EG ducks.
