# Supplementary material for: Escherichia coli O88 induces intestinal damage and inflammatory response through the oxidative phosphorylation and ribosome pathway in Pekin ducks
Source: Front Cell Infect Microbiol. 2022 Aug 17;12:940847. doi: 10.3389/fcimb.2022.940847 (PMC9433110; doi:10.3389/fcimb.2022.940847)
Supplement: Supplementary file 3 [file Table_1.docx]

**Additional file 1:**

| **Table S1** Analysis composition of basal diets and nutrient level (air-dry basis, %). | | |
| --- | --- | --- |
| Parameter | Starter (1 to 14 d) | Grower (14 to 28 d) |
| Ingredient |  |  |
| Corn | 58.90 | 67.98 |
| Soybean meal | 32.43 | 22.78 |
| Cottonseed meal | 2.00 | 4.00 |
| Soybean oil | 2.28 | 1.10 |
| Limestone | 1.07 | 1.14 |
| CaHPO4 | 1.71 | 1.49 |
| NaCl | 0.30 | 0.30 |
| *L*-Lys | 0.17 | 0.09 |
| *DL*-Met | 0.14 | 0.12 |
| Vitamin premix^1)^ | 0.03 | 0.03 |
| Microelement premix^2)^ | 0.10 | 0.10 |
| Zeolite powder | 0.87 | 0.87 |
| Total | 100.00 | 100.00 |
| Nutrient level^3)^ |  |  |
| Metabolizable energy (ME) (Mcal/Kg) | 2.90 | 2.90 |
| CP | 20.00 | 17.50 |
| Total lysine | 1.10 | 0.85 |
| Methionine | 0.45 | 0.40 |
| Total lysine + Methionine | 0.80 | 0.71 |
| Ca | 0.90 | 0.85 |
| Total P | 0.65 | 0.60 |
| Available P | 0.45 | 0.40 |

1. Amount provided per kilogram of diet: Vitamin A 10 000 IU, Vitamin D_3_ 2 000 IU, Vitamin E 10 IU, Vitamin K_3_ 2.5 mg, Vitamin B_1_ 1 mg, Vitamin B_2_ 6 mg, Vitamin B_3_ 10 mg, Vitamin B_5_ 40 mg, Vitamin B_6_ 3 mg, Vitamin B_11_ 0.3 mg, Vitamin B_12_ 0.01 mg, biotin 0.12 mg.
   2) The mineral premix provided the following per kilogram of diets: Cu (as copper sulfate) 8 mg, Fe (as ferrous sulfate) 80 mg, Mn (as manganese sulfate) 60 mg, Zn (as zinc sulfate) 40 mg, Se (as sodium selenite) 0.15 mg, I (as potassium iodide) 0.35 mg.
   3) Based on composition of ingredients provided by NY/T 2122-2012.

**Additional file 2:**

**Supplementary Figure S1** A KEGG map of the oxidative phosphorylation pathway of DEGs in CG vs EG. CG=control group, non-challenged with pathogenic *E. coli* O88; EG=experiment group, challenged with pathogenic *E. coli* O88. The boxes with green frames correspond to the down-regulation of DEGs in the EG ducks.

**Additional file 3:**

**Supplementary Figure S2** A KEGG map of the ribosomal pathway of DEGs in CG vs EG. CG=control group, non-challenged with pathogenic *E. coli* O88; EG=experiment group, challenged with pathogenic *E. coli* O88. The boxes with green frames correspond to the down-regulation of DEGs in the EG ducks.
